# Nutrition data use and needs: Findings from an online survey of global nutrition stakeholders

**DOI:** 10.7189/jogh.10.020403

**Published:** 2020-12

**Authors:** Audrey J Buckland, Andrew L Thorne-Lyman, Tricia Aung, Shannon E King, Renee Manorat, Laura Becker, Ellen Piwoz, Rahul Rawat, Rebecca Heidkamp

**Affiliations:** 1 Department of International Health, Johns Hopkins Bloomberg School of Public Health, Baltimore, Maryland, USA; 2 Results for Development, Washington, D.C., USA; 3 Bill & Melinda Gates Foundation, Seattle, Washington, USA

## Abstract

**Background:**

There is growing global demand for country-specific information to track nutritional status and its determinants, including intervention coverage. Periodic population-based surveys form the backbone of most national nutrition information systems. However, data on the coverage of many nutrition specific and sensitive interventions remain sparse.

**Methods:**

An online survey was administered to the international nutrition community in 2018 through relevant listservs and professional networks to characterize their use of nutrition-related indicators and data sources. Respondents were asked about their professional background, access and use of specific indicators and data sources in the previous year, and unmet data needs. Results were tabulated by respondent characteristics and χ^2^ tests used for statistical testing.

**Results:**

Complete survey responses were received from 235 respondents, the majority from non-governmental organizations and research communities, and few from governments. Demographic Health Surveys (DHS) were the most frequently accessed country-specific data source and the Global Nutrition Report (GNR) was the most accessed consolidated data source, each accessed by approximately 75% of respondents. Respondents with a multi-country focus were more likely to have accessed DHS than those with a single-country focus (85% vs 60%, *P* < 0.001). Similarly, respondents with a multi-country focus were more likely to have accessed the GNR compared to those with a single-country focus (82% vs 66%, *P* < 0.05). The most commonly accessed indicators overall were the prevalence of exclusive breastfeeding (69%), child minimum dietary diversity (66%), under-5 stunting (65%), and under-5 wasting (65%). Reported data gaps included adult and household diet quality indicators (n = 32), nutrition-sensitive intervention coverage (n = 25), and infant and young child feeding promotion coverage (n = 11). Lack of data availability for the desired geographic level (82%) or demographic group of interest (82%) and out-of-date data (77%) were common data challenges experienced by respondents.

**Conclusions:**

The survey results highlight the continued need for high-quality, actionable nutrition data to help facilitate progress towards national and global nutrition targets.

There is growing global demand for country-specific information to track population-level nutritional status and its determinants, including the coverage of key nutrition interventions. The 2014 Global Nutrition Report (GNR) called for a “nutrition data revolution” that includes filling data gaps and making information accessible in order to improve accountability and identify key areas for action [1]. The Sustainable Development Goal (SDG) to end malnutrition in all its forms by 2030 [2] reinforces the need for high-quality actionable nutrition data to track actions and progress on meeting this SDG.

Periodic population-based surveys form the backbone of most national nutrition information systems. Commonly available household surveys include Demographic and Health Surveys (DHS), the Multi-Indicator Cluster Surveys (MICS), as well as a variety of national and sub-national surveys, including the Standardised Monitoring and Assessment of Relief and Transitions (SMART), micronutrient and dietary consumption surveys. Many countries and development partners are also investing in strengthening routine facility data systems for nutrition by adding indicators to their District Health Information System 2 (DHIS-2) [3-5] and other sector-specific management information systems. However, data on the coverage of many nutrition specific and sensitive interventions remain sparse [6]. Intervention coverage is the proportion of the population who received a service out of those who were eligible. A review of the DHS and MICS core questionnaires – a set of survey questions implemented in every country – found that only 8 of the 23 WHO-recommended nutrition interventions are captured by these tools [7].

Adding new indicators to multi-topic household surveys (eg, DHS, MICS) and administrative systems is a resource-intensive process. It increases the data collection, training, and supervision burden and can compromise data quality due to data collector and/or respondent fatigue. Changes to administrative systems often require printing and distributing new registers to facilities, retraining of staff and changes to data tabulation and analysis. To justify these investments, we need clear evidence that potential users demand the information. To help characterize that demand, we conducted an online survey among nutrition professionals working in low- and middle- income countries (LMIC). The survey had two aims: 1) Identify the nutrition indicators and data sources currently used by stakeholder groups; 2) Identify the unmet nutrition information needs of these data users.

## METHODS

### Data collection

The online survey was created using Qualtrics software (Qualtrics, Provo, UT, USA), Version June 2018. The goal of our sampling was to get as many participants from the broader nutrition community as possible at all levels, and so we disseminated the survey through as many professional networks as possible. These included online nutrition-focused listservs with self-elected members (ie, Ag2Nut, Ag2Nut Ethiopia, CORE Group Nutrition, CORE Group General), institutional listservs (ie, WHO Nutritionlist and SUN), professional networks (ie, SUN, UNICEF, Bill & Melinda Gates Foundation, Johns Hopkins University), and Twitter. In this convenience sample, potential respondents were first contacted through an introductory email from the Data for Decisions to Expand Nutrition Transformation (DataDENT) study team, then were directed to a web-based portal in Qualtrics. Data were collected from July 16 to August 16, 2018.

Respondents were asked about their professional background, how they use data in their work, which nutrition indicators and data sources they accessed or used in the previous year as well as unmet data needs. Respondents were asked to identify which country-specific and consolidated data sources they accessed in the last 12 months. Consolidated data sources are those that present secondary data from multiple countries, typically using multiple data sources (eg, GNR, UNICEF State of the World’s Children Report, Scaling up Nutrition Monitoring, Evaluation, Accountability, and Learning (SUN MEAL)). Several questions allowed for multiple responses. Additional follow-up questions were asked about the data sources used by respondents that reported use of coverage data related to common interventions implemented in many countries, including routine growth monitoring for children, screening for acute malnutrition, treatment for severe acute malnutrition (SAM) or moderate acute malnutrition (MAM), vitamin A supplementation, iron folic acid (IFA) supplementation for women or adolescent girls, multiple micronutrient supplementation for women or adolescent girls, breastfeeding counseling, and complementary feeding counseling. The survey tool is available in Appendix S1 of the **Online Supplementary Document**.

Results were tabulated and compared across categories of geographic focus (single vs multi-country) and organization using Pearson χ-2 statistics calculated in Stata Version 14.0 (Stata Corporation, College Station, TX, USA). Respondents were not required to respond to all questions and therefore the sample size varies by question. The survey received a “Not Human Subjects Research” determination by the Johns Hopkins Bloomberg School of Public Health Institutional Review Board.

## RESULTS

### Respondent characteristics

A total of 264 survey responses were received; 235 with responses providing information beyond the identifiers section. The majority of respondents were from NGO and research communities, with relatively few respondents working in government (**Table 1**). The sample was highly educated and experienced, with over 90% of respondents having a master’s or doctoral degree. Two-thirds of respondents made nutrition-related monitoring and evaluation decisions in their professional roles. Of the 84% of respondents who self-identified as a technical expert on nutrition related issues, the most common domains of expertise included infant and young child feeding (IYCF) (74%), child nutrition (71%), and maternal nutrition (59%).

**Table 1:** Respondent background characteristics (N = 235)

Characteristic	No. (%)
**Organization:**	
Non-governmental organization (NGO)	70 (30%)
United Nations or other multinational agency	57 (24%)
University/research institute	54 (23%)
Government	27 (11%)
Donor	13 (6%)
Private sector	12 (5%)
Other	2 (1%)
**Educational achievement:**	
Secondary school	1 (0.4%)
Undergraduate	19 (8%)
Masters	130 (55%)
Doctoral	83 (35%)
Other	2 (1%)
**Years of experience:**	
0-1	7 (3%)
2-4	38 (16%)
5-9	65 (28%)
10+ years	125 (53%)
**Decisions made related to nutrition (multiple responses allowed):**	
Monitoring & evaluation	154 (66%)
Advocacy priorities	94 (40%)
Strategic program and policy planning	90 (38%)
Implementation	81 (34%)
Program-specific financial management	51 (22%)
Program administration	48 (20%)
High-level financing	30 (13%)
Other	27 (11%)
Technical support	8 (3%)
Research	5 (2%)

### Geographic work focus of respondents

The survey sample was evenly split between those who worked in a single country (49%) vs across multiple countries (51%). Among those who worked in a single country, half said their primary work focus was at the national level and half at the subnational level. Among those who worked across multiple countries, 40% worked at a global level, 25% at a regional level (eg, North Africa, Southeast Asia), 22% at a national level, and 14% at a subnational level (eg, state, district). Respondents were asked to list all the countries to which their work related in the last year, totaling 116 unique countries. By WHO region, the majority of respondents worked in at least one African country (63%), followed by South-East Asia (40%), the Americas (19%), the Eastern Mediterranean (17%), the Western Pacific (16%), and Europe (6%). The most frequently cited countries were India (n = 57), Ethiopia (n = 56), Kenya (n = 44), Bangladesh (n = 44), and Burkina Faso (n = 37).

### Data for decision-making

When asked about their current role working with data, 72% reported that they make decisions using data analyzed by others, 63% consolidate or analyze data for decision making within their institution, and 49% consolidate or analyze data for external decision making. Decisions made using these data varied by single and multiple country focus. Respondents working in a single country were more likely to make decisions related to program administration compared to those who worked in multiple countries (25% vs 15%, *P* = 0.0499). Those with a multicountry focus were more likely than single-country focus respondents to make strategic program and policy planning decisions (49% vs 26%, *P* < 0.001) or high-level financing decisions (18% vs 7%, *P* = 0.014).

### Access to country-specific and consolidated sources of data

Most respondents (81%) accessed at least one country-specific data source (eg, national household surveys, routine facility data systems, surveillance systems) in the last 12 months. Of those who accessed country-specific data in the last year, the DHS [8] were by far the most common (74%), followed by the MICS (42%) (**Table 2**). Respondents with a multi-country focus were more likely to access the DHS in the last year (85%) compared to those with a single-country focus (60%) (*P* < 0.001), a pattern that was even more apparent for the MICS (65% vs 16%, *P* < 0.001). Routine facility data sources such as the DHIS-2 and Health Management Information System (HMIS) were less accessed compared to household surveys, accessed only by about 30% of respondents, with no meaningful difference by respondents working at single vs multiple country levels. However, the DHIS-2 data was accessed more by single-country respondents working at a national level than those working at a subnational level (40% vs 21%, *P* = 0.048). The Service Provision Assessment (SPA), a health facility survey, was only accessed by one in ten respondents (11%).

**Table 2:** Country-specific data sources accessed in the last year by geographic level of focus, multiple responses allowed (n = 190)

Country-specific data sources	Geographic scope		
**Single country (N = 88)**	**Multi country (N = 102)**	**Total (N = 190)**
	**No. (%)**	**No. (%)**	**No. (%)**
Demographic Health Survey (DHS)	**53 (60%)**	87 (85%)*	140 (74%)
Multiple Indicator Cluster Survey (MICS)	**14 (16%)**	66 (65%)*	80 (42%)
Other National Nutrition Survey (eg, micronutrient survey)	39 (44%)	39 (38%)	78 (41%)
National survey using SMART methodology	**26 (30%)**	49 (48%)*	75 (39%)
National Dietary Intake / Food Consumption Survey	33 (38%)	31 (30%)	64 (34%)
Sub-national survey using SMART methodology	23 (26%)	39 (38%)	62 (33%)
DHIS-2 / similar online HMIS portal	29 (33%)	32 (32%)	61 (32%)
Health Management Information System (HMIS)	23 (26%)	30 (29%)	53 (28%)
Household, Income, Consumption & Expenditure survey	17 (19%)	18 (18%)	35 (18%)
National food security “hot spot” monitoring system/FEWS-NET	14 (16%)	20 (20%)	34 (18%)
World Bank Living Standard Measurement Studies (LSMS)	4 (5%)	25 (25%)	29 (15%)
WFP Food Security Monitoring System (FSMS)	6 (7%)	20 (20%)	26 (14%)
Other survey specific to program or policy	11 (13%)	13 (13%)	24 (13%)
WFP Comprehensive Food Security and Vulnerability Assessments (CFSVA)	6 (7%)	17 (17%)	23 (12%)
Other national household surveys with nutrition data	11 (13%)	10 (10%)	21 (11%)
Service Provision Assessment (SPA)	6 (7%)	15 (15%)	21 (11%)
WFP Emergency Food Security Assessment (EFSA)	6 (7%)	13 (13%)	19 (10%)
Demographic surveillance sites (DSS)	12 (14%)	7 (7%)	19 (10%)
Other facility survey	9 (10%)	7 (7%)	16 (8%)
Other national surveillance system	4 (5%)	6 (6%)	10 (5%)
Education MIS	6 (7%)	4 (4%)	10 (5%)
WASH MIS	6 (7%)	2 (2%)	8 (4%)
Agriculture MIS	2 (2%)	1 (1%)	3 (2%)
Other sector data systems	1 (1%)	3 (3%)	4 (2%)
Other national data sources	0 (0%)	2 (2%)	2 (1%)

*χ-2, *P* < 0.05, calculated for data sources used by at least 15% of respondents.

Most respondents accessed at least one source of consolidated data in the last 12 months (75%), of which the most common was the GNR [9] (**Table 3**). The GNR was more likely to be accessed by data users with a multi-country focus (82%) than those with a single-country focus (66%) (*P* = 0.014). Multi-country focused respondents were also more likely to access the UNICEF State of the World’s Children Report, other UNICEF nutrition data sets, and Countdown to 2030 data products (all *P* < 0.05).

**Table 3:** Consolidated data sources accessed in the last year by geographic level of focus, multiple responses allowed (n = 176)

Consolidated data sources	Geographic scope		
**Single country (N = 76)**	**Multi country (N = 100)**	**Total (N = 176)**
	**No. (%)**	**No. (%)**	**No. (%)**
Global Nutrition Report	**50 (66%)**	82 (82%)*	132 (75%)
UNICEF State of the World’s Children Report	**32 (42%)**	68 (68%)*	100 (57%)
UNICEF, WHO and the World Bank Joint Malnutrition Estimates	**22 (29%)**	47 (47%)*	69 (39%)
Other UNICEF Nutrition data sets	**21 (28%)**	46 (46%)*	67 (38%)
FAO The State of Food security and Nutrition in the World	23 (30%)	40 (40%)	63 (36%)
World Bank Nutrition Country Profiles	23 (30%)	39 (39%)	62 (35%)
Scaling up Nutrition Monitoring, Evaluation, Accountability and Learning (SUN MEAL)	25 (33%)	32 (32%)	57 (32%)
WHO Global Targets Tracking Tool	18 (24%)	33 (33%)	51 (29%)
Countdown to 2030	**16 (21%)**	35 (35%)*	51 (29%)
WHO Global Health Observatory	16 (21%)	27 (27%)	43 (24%)
FAO Country Indicators	11 (14%)	24 (24%)	35 (20%)
WHO Vitamin & Mineral Nutrition Information Systems	10 (13%)	22 (22%)	32 (18%)
WHO/UNICEF Joint Monitoring Programme for Water Supply, Sanitation and Hygiene	3 (4%)	22 (22%)	25 (14%)
IHME Global Burden of Disease	4 (5%)	20 (20%)	24 (14%)
Hunger and Nutrition Commitment Index Global: Country profiles	6 (8%)	14 (14%)	20 (11%)
FAO/WHO Global Individual Food Consumption Data Tool (GIFT)	5 (7%)	14 (14%)	19 (11%)
IHME Child Growth Failure	1 (1%)	10 (10%)	11 (6%)
Other global sources	1 (1%)	4 (4%)	5 (3%)

* χ-2, *P* < 0.05, calculated for data sources used by at least 15% of respondents.

### Access to specific types of indicators

**Figure 1:**
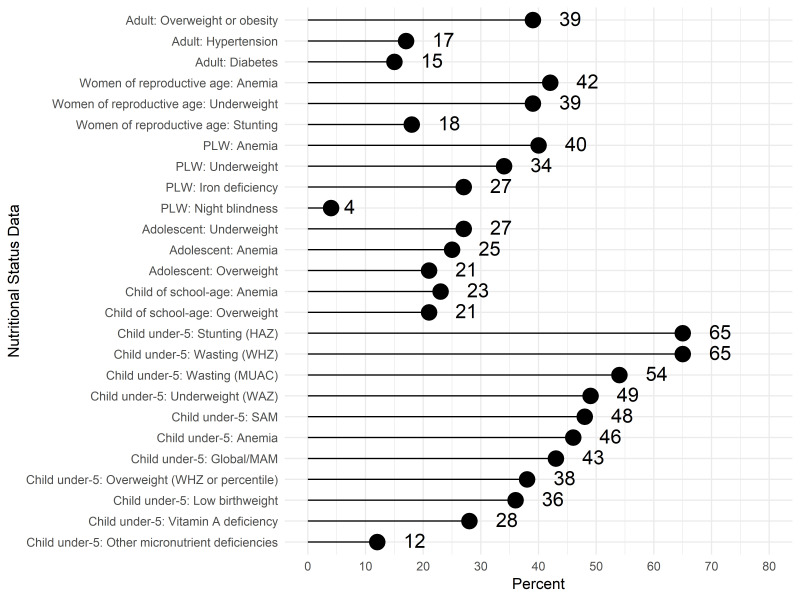
Access or use of nutritional status data (N = 235).

**Figure 2:**
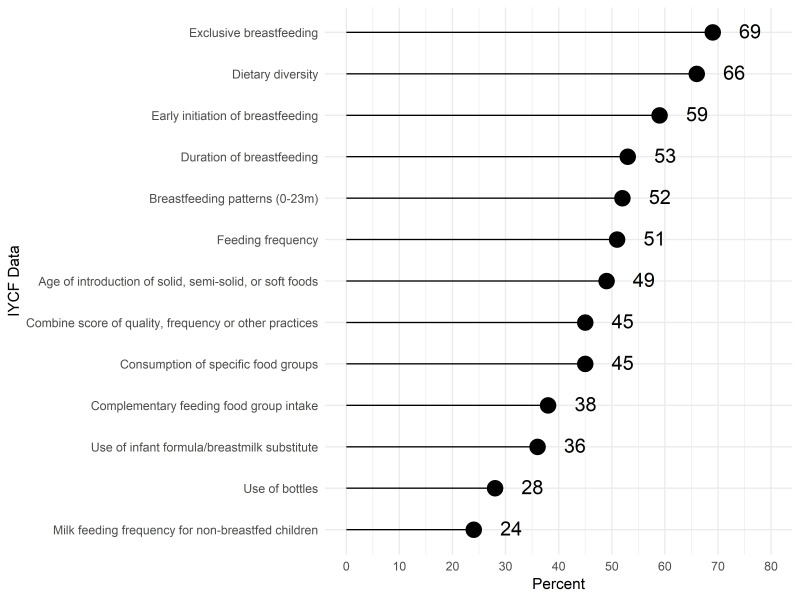
Access or use of infant and young child feeding practices data (N = 235).

**Figure 3:**
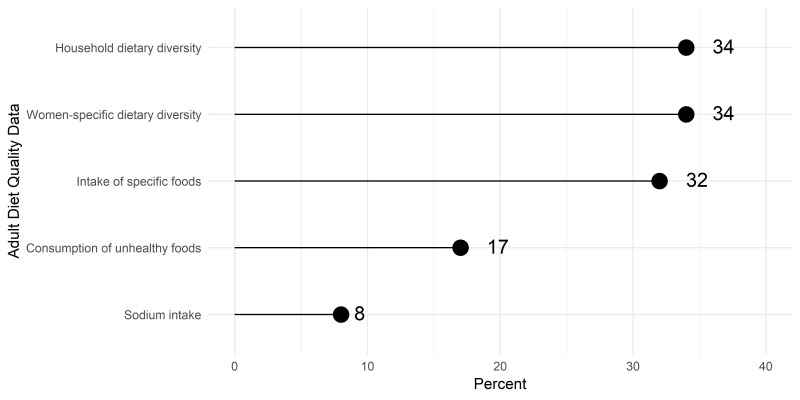
Access or use of adult diet quality data (N = 235).

When asked about types of data on nutritional status accessed in the previous 12 months, the most frequent responses were child anthropometric indicators, with the majority accessing data on child stunting and wasting (**Figure 1**). In comparison, fewer respondents accessed data on the nutritional status of other populations (adolescents, adults and school children). Data on IYCF practices, including breastfeeding and dietary diversity, were also accessed by most respondents (**Figure 2**). In contrast, less than a third of respondents accessed data related to the diets of women and adolescents (**Figure 3**).

**Figure 4:**
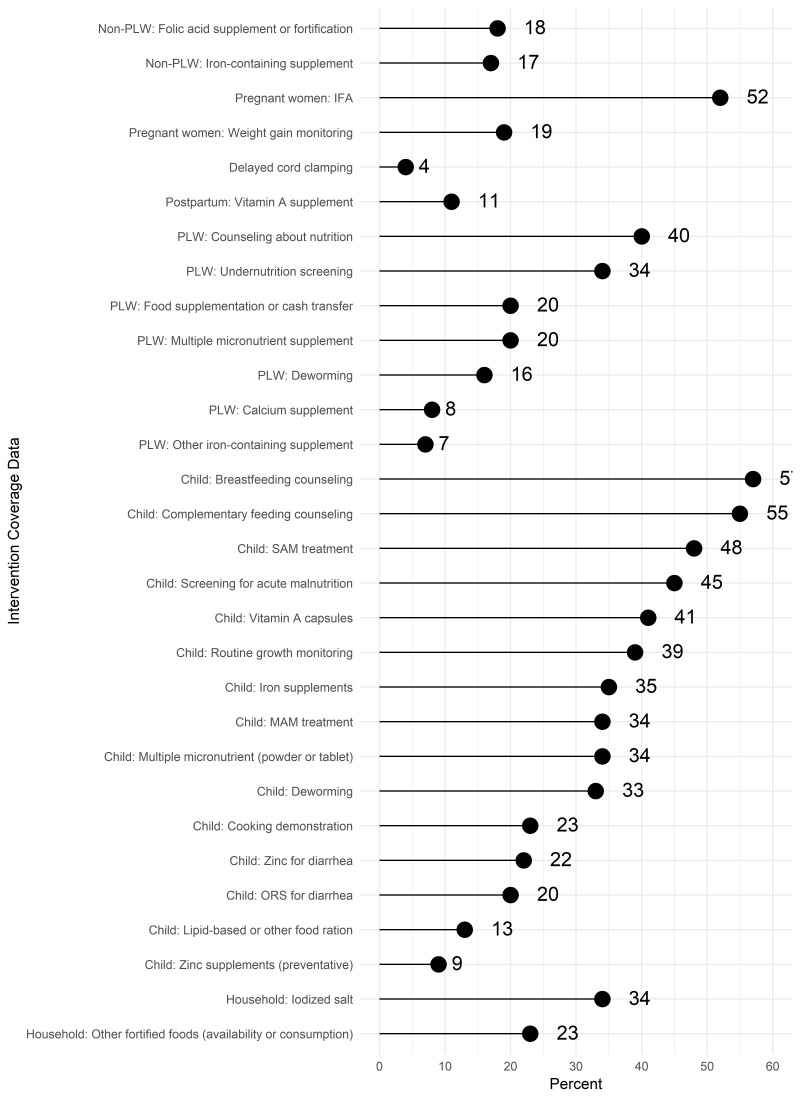
Access or use of coverage or utilization data (N = 235).

For intervention coverage and utilization indicators, more than half of respondents reported accessing data on coverage of breastfeeding counseling and complementary feeding counseling (**Figure 4**). Household surveys (69%) and administrative data (39%) were the most common sources reported by those who accessed breastfeeding counseling indicators (**Table 4**). The same two sources were reported at similar levels for complementary feeding counseling data. About half of those accessing breastfeeding or complementary feeding counseling coverage data wanted these data collected annually.

**Table 4:** Data sources accessed and desired availability for coverage indicators

	Data sources accessed,	No. (%)	Data sources considered “official”	No. (%)	Data are available at the frequency needed?	No. (%)	How frequently would you prefer data were collected, if you think it’s not available at the right frequency?	No. (%)
Growth monitoring (n = 92)	Household survey	63 (68%)	Household survey	68 (74%)	Yes	37 (40%)	2-5 y	9 (18%)
	Health facility survey	36 (39%)	Health facility survey	27 (29%)			Annually	15 (31%)
	Surveillance system	23 (25%)	Surveillance system	23 (25%)	No	49 (53%)	Quarterly	9 (18%)
	Administrative data	49 (53%)	Administrative data	44 (48%)			Monthly	8 (16%)
	Other	10 (11%)	Other	4 (4%)	Missing	6 (7%)	Other	5 (10%)
	Missing	6 (7%)	Missing	6 (7%)			Missing	3 (6%)
Acute malnutrition screening (n = 105)	Household survey	66 (63%)	Household survey	77 (73%)	Yes	53 (50%)	2-5 y	6 (12%)
	Health facility survey	26 (25%)	Health facility survey	19 (18%)			Annually	10 (20%)
	Surveillance system	34 (32%)	Surveillance system	26 (25%)	No	49 (47%)	Quarterly	15 (31%)
	Administrative data	57 (54%)	Administrative data	49 (47%)			Monthly	11 (22%)
	Other	15 (14%)	Other	7 (7%)	Missing	3 (3%)	Other	6 (12)
	Missing	5 (5%)	Missing	4 (4%)			Missing	1 (2%)
Preventive vitamin A capsules (n = 96)	Household survey	65 (68%)	Household survey	66 (69%)	Yes	64 (67%)	2-5 y	2 (8%)
	Health facility survey	17 (18%)	Health facility survey	14 (15%)			Annually	9 (36%)
	Surveillance system	14 (15%)	Surveillance system	18 (19%)	No	25 (26%)	Quarterly	6 (24%)
	Administrative data	55 (57%)	Administrative data	56 (58%)			Monthly	4 (16%)
	Other	6 (6%)	Other	7 (7%)	Missing	7 (7%)	Other	2 (8%)
	Missing	5 (5%)	Missing	5 (5%)			Missing	2 (8%)
Breastfeeding counseling (n = 134)	Household survey	93 (69%)	Household survey	90 (67%)	Yes	46 (34%)	2-5 y	11 (13%)
	Health facility survey	29 (22%)	Health facility survey	22 (16%)	No	82 (61%)	Annually	41 (50%)
	Surveillance system	16 (12%)	Surveillance system	19 (14%)			Quarterly	15 (18%)
	Administrative data	52 (39%)	Administrative data	56 (42%)			Monthly	12 (15%)
	Other	19 (14%)	Other	13 (10%)			Other	3 (4%)
	Missing	8 (6%)	Missing	12 (9%)	Missing	6 (4%)	Missing	0 (0%)
Complementary feeding counseling (n = 129)	Household survey	94 (73%)	Household survey	93 (72%)	Yes	44 (34%)	2-5 y	13 (16%)
	Health facility survey	20 (16%)	Health facility survey	20 (16%)	No	80 (62%)	Annually	38 (48%)
	Surveillance system	21 (16%)	Surveillance system	22 (17%)			Quarterly	14 (18%)
	Administrative data	45 (35%)	Administrative data	47 (36%)			Monthly	13 (16%)
	Other	16 (12%)	Other	13 (10%)			Other	2 (3%)
	Missing	8 (6%)	Missing	9 (7%)	Missing	5 (4%)	Missing	0 (0%)

Half of the respondents reported accessing data on IFA supplementation during pregnancy (**Figure 4**). The specific IFA indicators used or accessed varied: any IFA consumed (77%), minimum number of tablets consumed (58%), IFA purchased or received (49%).

**Figure 5:**
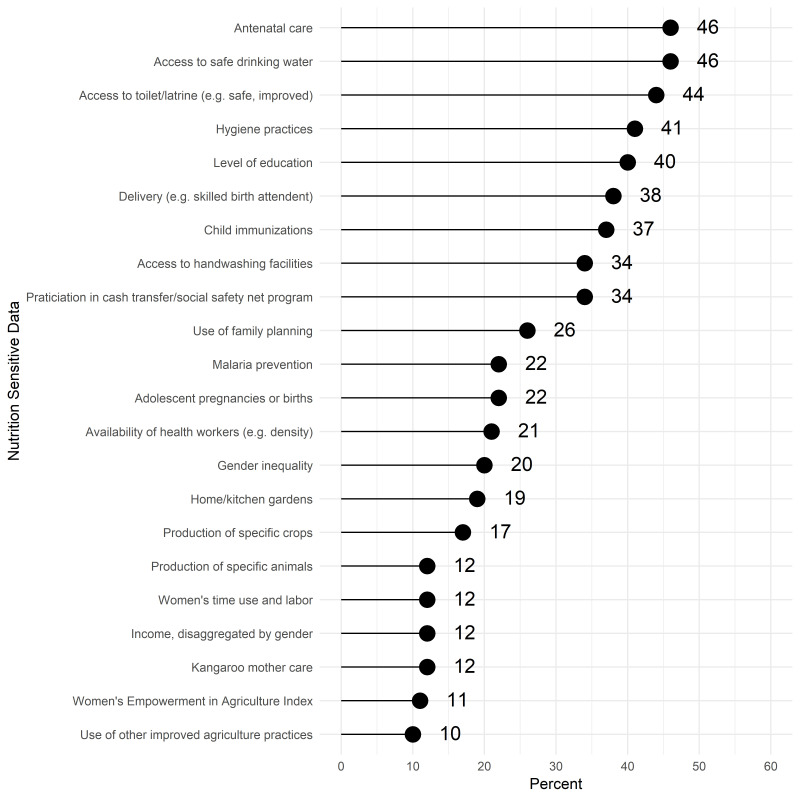
Access or use of nutrition sensitive interventions or drivers data (N = 235).

About half of respondents reported accessing data on screening for acute malnutrition and/or treatment of SAM. More respondents reported accessing screening data from household surveys than administrative data. In contrast for SAM or MAM treatment, administrative data sources were most accessed (**Table 4**). For both screening and treatment, about half of respondents who reported accessing the indicators in the last year were satisfied with the current periodicity of available data. The most commonly accessed or used data on nutrition-sensitive interventions were data on water, sanitation and hygiene (**Figure 5**).

Compared to respondents with a single-country focus, those with a multi-country focus were more likely to access indicators for under-5 wasting, overweight, and vitamin A deficiency, adolescent overweight, women of reproductive age stunting, underweight, and anemia, and gender inequality (all *P* < 0.05).

### Challenges to accessing and using data

**Figure 6:**
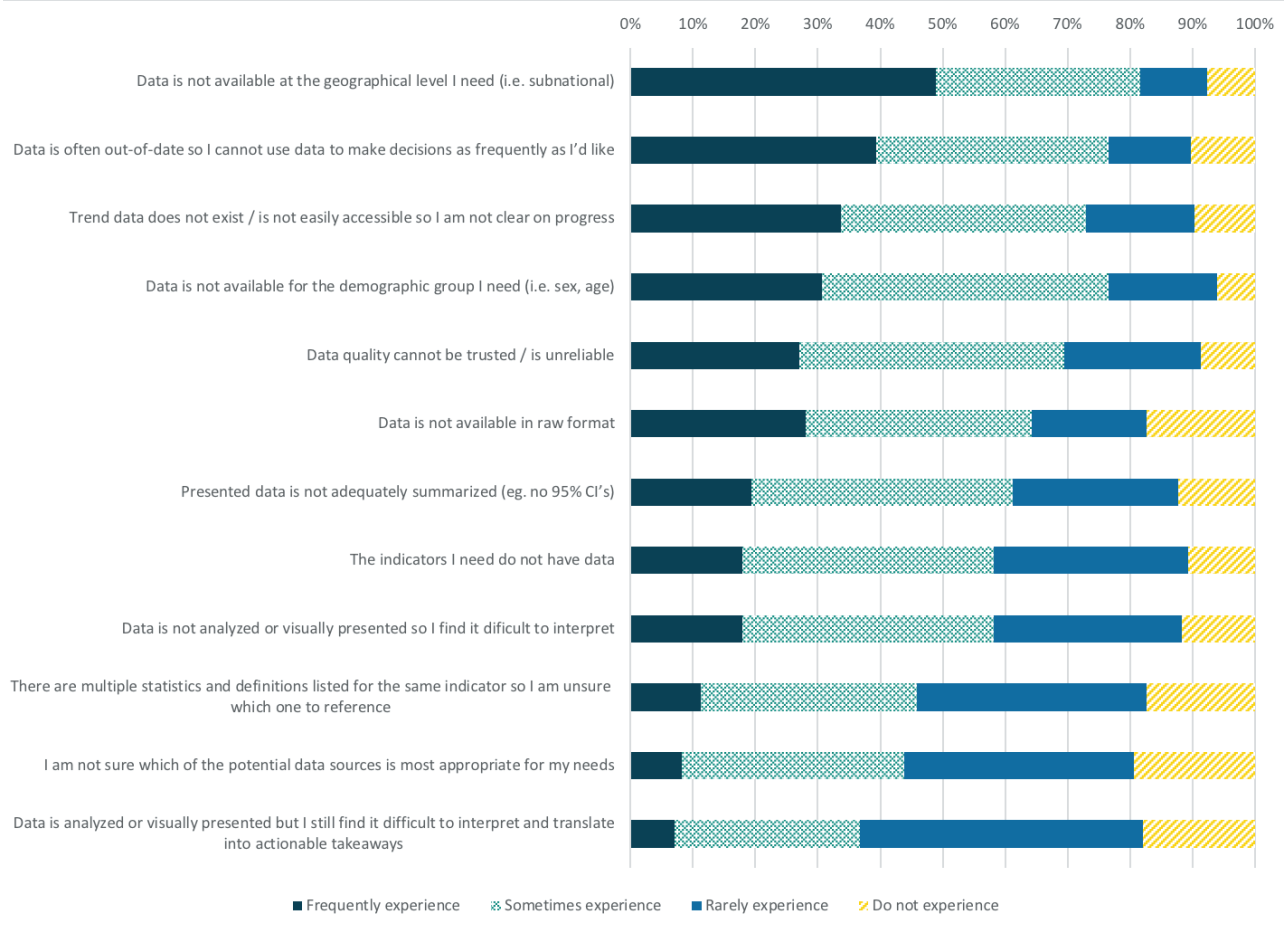
Challenges experienced in accessing and using nutrition data (N = 196).

Several challenges to accessing and using nutrition data were reported by respondents. Most respondents reported that data were sometimes or frequently not available at the geographical disaggregation needed (82%), for the demographic group needed (77%), or were out-of-date (77%) (**Figure 6**). There were no significant differences between multi-country and single-country focus for reported challenges. Responses to an open-ended question about data gaps identified nutrition-sensitive intervention coverage, IYCF promotion and counseling coverage data as priorities.

## DISCUSSION

To our knowledge, this is the first published survey of perceptions of nutrition data use and gaps among stakeholders who work globally in nutrition. Both for stakeholders working in a single country and across multiple countries, the most important data sources were the DHS and the GNR. It is not surprising that the DHS is the most commonly accessed country-specific data source as the DHS program is implemented in more than 90 countries and continues to be a staple for the global health community [10]. We expected respondents with a single-country focus to access the DHIS-2 or HMIS more than those working across multiple countries, but there was no difference. This could be explained by our lack of respondents from LMIC governments. The DHIS-2 has been used in 67 LMICs, and it is possible that we were unable to capture these respondents. Additionally, there might have been more variable access to DHIS-2 or HMIS among respondents.

A surprising finding was that respondents reported accessing data on coverage of breastfeeding counseling and complementary feeding counseling at a much higher rate than we expected, particularly given that questions about the coverage of breastfeeding counseling were only recently added to the DHS and MICS and few countries have data on complementary feeding counseling coverage. It is possible that respondents confused indicators of IYCF counseling coverage with indicators of IYCF practices (eg, early initiation of breastfeeding, exclusive breastfeeding, minimum dietary diversity) which have been collected in DHS and MICS for many years and in some administrative systems.

Coverage of antenatal IFA supplementation is a core process indicator of the Global Nutrition Monitoring Framework (GNMF). The results of this survey showed that three different indicators are accessed for antenatal IFA supplementation – IFA consumed, minimum number of tablets consumed, and IFA purchased or received. In 2018, the WHO-UNICEF Technical Expert Advisory group on nutrition Monitoring (TEAM) proposed a simplified definition of antenatal IFA supplementation coverage to accommodate the variability in available data sources: “The percentage of women consuming any iron-containing supplements during the current or past pregnancy within the last 2 years” [11].

### Limitations

The generalizability of our survey findings to the broader global nutrition community is uncertain. The survey was disseminated broadly, using established listservs, institutional contacts, and Twitter. This study utilized convenience sampling; given our dissemination channels, those without reliable internet access and those who were not connected to the listservs or professional networks utilized may have been underrepresented. We had very few respondents from LMIC governments, a high priority user group. We also had very few respondents with less than a college education, perhaps suggesting under-representation of front-line workers. Further effort is needed to better understand how national and subnational government stakeholders access and use nutrition data.

## CONCLUSIONS AND FUTURE PRIORITIES

Our findings suggest that nutrition stakeholders have a strong demand for timely data, particularly on nutritional status and IYCF practices and suggest the need to continue and even expand investment in surveys that generate these indicators. Our findings also suggest strong demand for more frequent data collection and more data in general for program coverage indicators including growth monitoring, breastfeeding counseling, and complementary feeding counseling. At the same time, it is important to acknowledge that DHS and MICS multi-topic survey questionnaires are quite lengthy and it is difficult to collect additional indicators without compromising survey quality. This challenge raises questions about what new systems may be needed to support greater frequency of data collection and to potentially support demand for finer granularity in the representativeness of certain indicators at the subnational level.

While there have been ongoing efforts by WHO-UNICEF TEAM to standardize and validate indicator definitions and operational guidance, continued effort is needed, especially to refine data collection tools. These needs are being addressed in part by a number of nutrition-data focused initiatives including WHO-UNICEF TEAM, DataDENT, National Information Platforms for Nutrition, SUN MEAL, and Nutrition for Growth [7]. Finally, the data challenges point to a need to improve data literacy so users are better able to decide which data are most appropriate for their needs.

## Additional material

Online Supplementary Document
