# Supplementary material for: Nutrition data use and needs: Findings from an online survey of global nutrition stakeholders
Source: J Glob Health. 2020 Nov 8;10(2):020403. doi: 10.7189/jogh.10.020403 (PMC7688248; doi:10.7189/jogh.10.020403)
Supplement: Online Supplementary Document [file jogh-10-020403-s001.pdf]

Appendix S1. Questionnaire

Q#	Section A: Respondent background information		Skip
A	I agree to participate in the survey. I understand the purpose and nature of this activity and I am participating voluntarily. I understand that I can stop taking the survey at any time, without any penalty or consequences.	Yes ……………………………………………...………………………………………..…….1<br>No …………………………………………………………………………………………..…….2	
A1	What type of organization do you work for?	Government ……………………………...…………………………………………………………..1<br>UN or similar multinational agency (eg SUN, African Union) …………………………….…….2<br>NGO ……………………………………………………………………………………………….3<br>Donor (public or private) …………..…………………………………………………………….4<br>University/Research institute ……………..………………………………………………………5<br>Private Sector ……………………………………………………………………………………..6<br>Other- specify.……………………………………………………………………………………..7	
A2	What types of decisions related to nutrition do you make or support in your current professional role? Please check all that apply.	Implementation: *manage day-to-day programming* ………………………………….......………1<br>Program administration: *coordinate and manage program logistics* ……………………...…………..2<br>Monitoring & Evaluation: *monitor progress of policy or program implementation …………..……....3*<br>Program-specific financial management: *management of the financial resources within specific programs or projects ……………………..*……………………………………………………….4<br>Strategic program and policy planning*: Sets strategic vision and allocates resources for policies or programs ………….*…………………………………………………………………………….5<br>Advocacy priorities: *whether to raise awareness for a particular issue ………………………………...6*<br>High-level financing*: investment decisions for a donor, government or other institution ……………..7*<br>Other*:* (specify) ..……………………………………………………………………………….8	
A3	Do you consider yourself a technical expert on nutrition-related issues?	Yes ……………………………………………...………………………………………..…….1<br>No …………………………………………………………………………………………..…….2	→ A4b
A3b	What do you consider your areas of focus or expertise? Please check all that apply.	Infant and young child feeding (IYCF) ..…………………………………………………………1<br>Micronutrients ..…………………………………………………………………………………..2<br>Child nutrition ..…………………………………………………………………………………..3<br>Adolescent nutrition ..……………………………………………………………………………..4	

		Maternal nutrition ……5<br>Obesity and non-communicable diseases ……6<br>Food security and food systems ……7<br>Water, sanitation & hygiene (WASH) ……8<br>Humanitarian Emergencies ……9<br>Costing/cost effectiveness ……10<br>Other ……11	
A4	What is your highest education level achieved?	Secondary (high) school ……1<br>Undergraduate ……2<br>Masters ……3<br>Doctoral (e.g. PhD, MD) ……4<br>Other (specify) ……5	
A5	For how many years have you worked on nutrition-related issues?	0-1 years ……1<br>2-4 years ……2<br>5-9 years ……3<br>10+ years ……4	
A6	In the last 12 months, what has been the geographic scope of your nutrition-related work?	Within a single country ……1<br>Across multiple countries ……2	→ A9a<br>→A9b
A6a	Within that country, at what level are you primarily working? Please select one.	National ……1<br>Subnational (e.g. state, district) ……2	
A6b	Across those countries, what is your level of primary focus? Please select one.	Global ……1<br>Global regional (e.g. North Africa, Southeast Asia) ……2<br>National ……3<br>Subnational (e.g. state, district) ……4	
A7	In the past 12 months, which country or countries has your work related to?<br>Please select all that apply.	Africa:<br>• Algeria<br>• Angola<br>• Benin<br>• Botswana<br>• Burkina Faso<br>• Burundi<br>• Cameroon<br>• Cape Verde<br>• Central African Republic<br>• Chad<br>• Comoros<br>• Côte d'Ivoie<br>• Sao Tome and Principe<br>• Senegal<br>• Seychelles<br>• Sierra Leone<br>• South Africa<br>• Swaziland<br>• Togo<br>• Uganda<br>• United Republic of Tanzania<br>• Zambia<br>• Zimbabwe<br>Americas:<br>• Belize<br>South-East Asia:<br>• Bangladesh<br>• Bhutan<br>• India<br>• Indonesia<br>• Myanmar<br>• Nepal<br>• Sri Lanka<br>• Thailand<br>• Timor-Leste<br>Europe:<br>• Kazakhstan<br>• Kyrgyzstan	

		• Democratic Republic of the Congo<br>• Equatorial Guinea<br>• Eritrea<br>• Ethiopia<br>• Gabon<br>• Gambia<br>• Ghana<br>• Guinea<br>• Guinea-Bissau<br>• Kenya<br>• Lesotho<br>• Liberia<br>• Madagascar<br>• Malawi<br>• Mali<br>• Mauritania<br>• Mauritius<br>• Mozambique<br>• Namibia<br>• Niger<br>• Nigeria<br>• Republic of the Congo<br>• Rwanda<br>•	• Bolivia<br>• Colombia<br>• Costa Rica<br>• Cuba<br>• Dominica<br>• Dominican Republic<br>• Ecuador<br>• El Salvador<br>• Grenada<br>• Guatemala<br>• Guyana<br>• Haiti<br>• Honduras<br>• Jamaica<br>• Mexico<br>• Nicaragua<br>• Panama<br>• Paraguay<br>• Peru<br>• Uruguay<br>Venezuela	• Tajikistan<br>Eastern Mediterranean:<br>• Afghanistan<br>• Pakistan<br>• Somalia<br>• Sudan<br>• Syrian Arab Republic<br>Yemen<br>Western Pacific:<br>• Cambodia<br>• Fiji<br>• Lao People's Democratic Republic<br>• Marshall Islands<br>• Mongolia<br>• Papua New Guinea<br>• Philippines<br>• Viet Nam	
A7b	*Triggered if A10 is more than 3:* Which three countries do you consider the primary focus in your current work?<br><br>NOTE: Answer "NA" if no individual countries are given higher priority among those you selected in the previous question.	<free response line 1><br><free response line 2><br><free response line 3><br>Not Applicable (NA) - No individual countries are given more focus than others			

A8	Which of the following describes how your current role involves working with data? Please select all that apply.	I am directly involved in the collection of quantitative data through surveys, administrative systems, or other approaches …………..……………………………………………………………………..1<br>I manage or update a database or data repository ..……………………………………………………2<br>I consolidate-and/or analyze data from one or more sources for internal decision making (by myself or my team) ..………………………………………………………………………………………………3<br>I consolidate-and/or analyze data from one or more sources for external decision making (by others outside my team) ………………………………………………………………………………………4<br>I use data that has been consolidated and/or analyzed by others (e.g. in a report, presentation, or other format) for decision making ..………………………………………………………………………5	
	Section B: Indicator use (Indicator: a measure that provides information about a specifically defined element)		
B1	In the last 12 months have you accessed or used coverage / utilization data for any of the following interventions? Select all that apply.	**No - I have not accessed any data on coverage or utilization of nutrition interventions …………0**<br><br>**Child**:<br>Routine growth monitoring ..……………………………………………………………………………1<br>Screening for Acute Malnutrition ..…………………………………………………………………….2<br>ORS for diarrhea ..………………………………………………………………………………………3<br>Zinc as diarrhea treatment ..……………………………………………………………………………..4<br>Severe Acute Malnutrition (SAM) treatment ..…………………………………………………………5<br>Moderate Acute Malnutrition (MAM) treatment ..……………………………………………………..6<br>Vitamin A capsules ..……………………………………………………………………………………7<br>Deworming ..……………………………………………………………………………………………8<br>Multiple Micronutrient (Powder or Tablet) ..……………………………………………………………..9<br>Iron supplements ..……………………………………………………………………………………….10<br>Zinc supplements (preventative; NOT for diarrhea) ..…………………………………………………11<br>Provision of lipid-based supplement or other food ration ..……………………………………………...12<br>Breastfeeding counseling (for mother/caregiver) ..……………………………………………………13<br>Complementary Feeding Counseling (for mother/caregiver) ..………………………………………..14<br>Cooking demonstration ..………………………………………………………………………………15<br><br>**Women and/or adolescent girls**:<br>*Specific to pregnant and/or lactating*<br>Iron Folic Acid Supplementation ..……………………………………………………………………16<br>Multiple Micronutrient Supplementation ..……………………………………………………………17<br>Other iron-containing supplement ..……………………………………………………………………...18<br>Calcium supplementation ..……………………………………………………………………………19<br>Delayed cord clamping ..………………………………………………………………………………20<br>Post-partum Vitamin A supplement ..…………………………………………………………………21<br>Deworming ..……………………………………………………………………………………………..22<br>Counseling about nutrition during pregnancy or lactation ..……………………………………………..23<br>Monitoring of weight gain during pregnancy..…………………………………………………………...24	 <br><br><br>→B1c1<br>→B1d1<br><br>→B1e1<br>→B1e1<br><br>→B1f1<br><br><br><br><br><br>→B1g1<br>→B1h1<br><br><br><br><br>→B1a<br>→B1b

		Screening for undernutrition (e.g. low MUAC/BMI) ..……………………………………………..25<br>Food supplementation or cash transfer ..……………………………………………………………26<br><br>*For other women or adolescents (non-pregnant / non-lactating)*<br>Iron–containing supplement ..…………………………………….. ..…………………………...27<br>Folic acid supplementation or fortification ..………………………………………………………..28<br><br>**Household**:<br>Iodized salt ..……………………………………..………………………………………………..29<br>Other fortified foods – availability or consumption ..…………………………………………………30	 <br><br><br>→B1j & B1l<br>→B1k & B1l
B1a:	Which of the following indicators related to IFA did you access or use? Please select all that apply.	IFA purchased or received ……………………………………...………………………………1<br><br>IFA consumed ……………………………………………………………..……………………...2<br><br>Minimum number of tablets consumed (e.g. at least 90) ……………………………………..……..3	
B1b:	Which of the following indicators related to Multiple Micronutrient Supplementation (MMN) did you access or use? Please select all that apply.	MMN purchased or received ……………………………………...……………………………1<br><br>MMN consumed ………………………………………………………….............................2<br><br>Minimum number of tablets consumed (e.g. at least 90) ……………………………………….....3	
B1c1	From what types of data source did you access growth monitoring data? Please select all that apply	Household survey (e.g. DHS/MICS/SMART/other household survey) …………………………..…...1<br>Health facility survey (e.g. SPA, other) ………………………………………....………………...2<br>Surveillance System (e.g. DSS, Hot Spot monitoring, etc.) ……………………….,……………......3<br>Administrative (routine) data source (e.g. DHIS-2, HMIS, other administrative data) ………………..4<br>Other (please specify) ……………………………………………………….....………………...5	
B1c2	In your work context, which of these data sources are considered the “official” / most often quoted for growth monitoring data? Please select all that apply.	Household survey (e.g. DHS/MICS/SMART/other household survey) …………………………..…...1<br>Health facility survey (e.g. SPA, other) ………………………………………....………………...2<br>Surveillance System (e.g. DSS, Hot Spot monitoring, etc.) ……………………….,……………......3<br>Administrative (routine) data source (e.g. DHIS-2, HMIS, other administrative data) ………………..4<br>Other (please specify) ……………………………………………………….....………………...5	

B1c3	Are new growth monitoring data available at a frequency/interval that meets your needs?	Yes ……………………………...……………………………………………..………...............................1<br>No ……2	→ B1c4
B1c4	How frequently would you prefer to have new growth monitoring data for your purposes?	Every 6-10 years ………………………………………………………………...……………….............1<br>Every 2-5 years ………………………………………………………………...……………….............2<br>Every year (annual) …………………………………………………………...………………….......3<br>Quarterly ………………………………………………………………...……………….......................4<br>Monthly ………………………………………………………………....……………….........................5<br>Other: Please specify ……………………………………………………………...…………………....6	
B1d1	From what types of data source did you access acute malnutrition screening data? Please select all that apply	Household survey (e.g. DHS/MICS/SMART/other household survey) ……………………………..…...1<br>Health facility survey (e.g. SPA, other) …………………………………………...…………………...2<br>Surveillance System (e.g. DSS, Hot Spot monitoring, etc.) ……………………….,…………….......3<br>Administrative (routine) data source (e.g. DHIS-2, HMIS, other administrative data) ………………..4<br>Other (please specify) ……………………………………………………………...…………………...5	
B1d2	In your work context, which of these data sources are considered the "official" / most often quoted for acute malnutrition screening data? Please select all that apply.	Household survey (e.g. DHS/MICS/SMART/other household survey) ……………………………..…...1<br>Health facility survey (e.g. SPA, other) …………………………………………...…………………...2<br>Surveillance System (e.g. DSS, Hot Spot monitoring, etc.) ……………………….,…………….......3<br>Administrative (routine) data source (e.g. DHIS-2, HMIS, other administrative data) ………………..4<br>Other (please specify) ……………………………………………………………...…………………...5	
B1d3	Are new acute malnutrition screening data available at a frequency/interval that meets your needs?	Yes ……………………………...……………………………………………..………...............................1<br>No ……2	→ B1d4

B1d4	How frequently would you prefer to have new acute malnutrition screening data for your purposes?	Every 6-10 years ……………………………………………………………...……………….............1<br>Every 2-5 years ……………………………………………………………...……………….............2<br>Every year (annual) ……………………………………………………...……………….......3<br>Quarterly ……………………………………………………………...………………........................4<br>Monthly ……………………………………………………………...………………........................5<br>Other: Please specify ……………………………………………………...…………………....6	
B1e1	From what types of data source did you access Severe Acute Malnutrition (SAM) or Moderate Acute Malnutrition (MAM) treatment data? Please select all that apply	Household survey (e.g. DHS/MICS/SMART/other household survey) …………………………...…...1<br>Health facility survey (e.g. SPA, other) …………………………………………...…………………...2<br>Surveillance System (e.g. DSS, Hot Spot monitoring, etc.) ………………………..……………......3<br>Administrative (routine) data source (e.g. DHIS-2, HMIS, other administrative data) ……………….4<br>Other (please specify) ……………………………………………………...…………………...5	
B1e2	In your work context, which of these data sources are considered the "official" / most often quoted for Severe Acute Malnutrition (SAM) or Moderate Acute Malnutrition (MAM) treatment data? Please select all that apply.	Household survey (e.g. DHS/MICS/SMART/other household survey) …………………………...…...1<br>Health facility survey (e.g. SPA, other) …………………………………………...…………………...2<br>Surveillance System (e.g. DSS, Hot Spot monitoring, etc.) ………………………..……………......3<br>Administrative (routine) data source (e.g. DHIS-2, HMIS, other administrative data) ……………….4<br>Other (please specify) ……………………………………………………...…………………...5	
B1e3	Are Severe Acute Malnutrition (SAM) or Moderate Acute Malnutrition (MAM) treatment data available at a frequency/interval that meets your needs?	Yes ………………………...…………………………………………..………..............................1<br>No ………………………………………………………………………………………………2	 → B1e4

B1e4	How frequently would you prefer to have new Severe Acute Malnutrition (SAM) or Moderate Acute Malnutrition (MAM) treatment data for your purposes?	Every 6-10 years ……………………………………………………………...……………….........1<br>Every 2-5 years ……………………………………………………………...……………….............2<br>Every year (annual) ……………………………………………………………...……………….......3<br>Quarterly ……………………………………………………………...……………….........................4<br>Monthly ……………………………………………………………...……………….........................5<br>Other: Please specify ……………………………………………………………...…………………...6	
B1f1	From what types of data source did you access preventative Vitamin A capsules coverage data? Please select all that apply	Household survey (e.g. DHS/MICS/SMART/other household survey) ………………………...…...1<br>Health facility survey (e.g. SPA, other) ……………………………………………...………………...2<br>Surveillance System (e.g. DSS, Hot Spot monitoring, etc.) ………………………,……………......3<br>Administrative (routine) data source (e.g. DHIS-2, HMIS, other administrative data) ………………..4<br>Other (please specify) ……………………………………………………………...…………………..5	
B1f2	In your work context, which of these data sources are considered the "official" / most often quoted for preventative Vitamin A capsules coverage data? Please select all that apply.	Household survey (e.g. DHS/MICS/SMART/other household survey) ………………………...…...1<br>Health facility survey (e.g. SPA, other) ……………………………………………...………………...2<br>Surveillance System (e.g. DSS, Hot Spot monitoring, etc.) ………………………,……………......3<br>Administrative (routine) data source (e.g. DHIS-2, HMIS, other administrative data) ………………..4<br>Other (please specify) ……………………………………………………………...…………………..5	
B1f3	Are new preventative Vitamin A capsules coverage data available at a frequency/interval that meets your needs?	Yes ………………………...…………………………………………...………...............................1<br>No ………………………………………………………………………………………………………2	 → B1f4
B1f4	How frequently would you prefer to have new preventative Vitamin A capsules coverage data for your purposes?	Every 6-10 years ……………………………………………………………...……………….........1<br>Every 2-5 years ……………………………………………………………...……………….............2<br>Every year (annual) ……………………………………………………………...……………….......3<br>Quarterly ……………………………………………………………...……………….........................4<br>Monthly ……………………………………………………………...……………….........................5<br>Other: Please specify ……………………………………………………………...…………………...6	

B1g1	From what types of data source did you access breastfeeding counselling coverage data? Please select all that apply	Household survey (e.g. DHS/MICS/SMART/other household survey) ……………………………..…...1<br>Health facility survey (e.g. SPA, other) ………………………………………………...………………...2<br>Surveillance System (e.g. DSS, Hot Spot monitoring, etc.) ……………………….,…………….......3<br>Administrative (routine) data source (e.g. DHIS-2, HMIS, other administrative data) ………………..4<br>Other (please specify) …………………………………………………………...………………...5	
B1g2	In your work context, which of these data sources are considered the "official" / most often quoted for breastfeeding counselling coverage data? Please select all that apply.	Household survey (e.g. DHS/MICS/SMART/other household survey) ……………………………..…...1<br>Health facility survey (e.g. SPA, other) ………………………………………………...………………...2<br>Surveillance System (e.g. DSS, Hot Spot monitoring, etc.) ……………………….,…………….......3<br>Administrative (routine) data source (e.g. DHIS-2, HMIS, other administrative data) ………………..4<br>Other (please specify) …………………………………………………………...………………...5	
B1g3	Are new breastfeeding counselling coverage data available at a frequency/interval that meets your needs?	Yes ………………………...……………………………………………..………..............................1<br>No ……………………………………………………………………………………………………2	→ B1g4
B1g4	How frequently would you prefer to have new breastfeeding counselling coverage data for your purposes?	Every 6-10 years ……………………………………………………………...……………….........1<br>Every 2-5 years ……………………………………………………………...………………............2<br>Every year (annual) ……………………………………………………...…………………........3<br>Quarterly ………………………………………………………………..……………….......................4<br>Monthly ……………………………………………………………...……………….........................5<br>Other: Please specify ……………………………………………………………...………………....6	
B1h1	From what types of data source did you access complementary feeding counseling coverage data? Please select all that apply	Household survey (e.g. DHS/MICS/SMART/other household survey) ……………………………..…...1<br>Health facility survey (e.g. SPA, other) ………………………………………………...………………...2<br>Surveillance System (e.g. DSS, Hot Spot monitoring, etc.) ……………………….,…………….......3<br>Administrative (routine) data source (e.g. DHIS-2, HMIS, other administrative data) ………………..4<br>Other (please specify) …………………………………………………………...………………...5	

B1h2	In your work context, which of these data sources are considered the "official" / most often quoted for complementary feeding counseling coverage data? Please select all that apply.	Household survey (e.g. DHS/MICS/SMART/other household survey) ……………………………..…...1<br>Health facility survey (e.g. SPA, other) …………………………………………...………………...2<br>Surveillance System (e.g. DSS, Hot Spot monitoring, etc.) ………………………..,……………......3<br>Administrative (routine) data source (e.g. DHIS-2, HMIS, other administrative data) ………………..4<br>Other (please specify) …………………………………………………………...…………………...5	
B1h3	Are new complementary feeding counseling coverage data available at a frequency/ interval that meets your needs?	Yes ………………………...…………………………………………..………................................1<br>No …………………………………………………………………………………………………2	→ B1h4
B1h4	How frequently would you prefer to have new complementary feeding counseling coverage data for your purposes?	Every 6-10 years …………………………………………………………...………………...........1<br>Every 2-5 years …………………………………………………………...………………..............2<br>Every year (annual) ………………………………………………………...………………........3<br>Quarterly ………………………………………………………………...………………........................4<br>Monthly ………………………………………………………………...……………….........................5<br>Other: Please specify ………………………………………………………...…………………....6	
B1j	For which age group do you access iron-containing supplements data?	Adolescents ……………………………………………………………...………………...................1<br>Women ………………………………………………………………...……………….........................2<br>Both adolescents and women ………………………………………………………...…………3	
B1k	For which age group do you access folic acid supplementation or fortification supplements data?	Adolescents ……………………………………………………………...………………...................1<br>Women ………………………………………………………………...……………….........................2<br>Both adolescents and women ………………………………………………………...…………3	

B1l	From what types of data source did you access non-pregnant, non-lactating supplementation data?<br>Please select all that apply	Household survey (e.g. DHS/MICS/SMART/other household survey) ……………………………..…...1<br>Health facility survey (e.g. SPA, other) ………………………………………………...………………...2<br>Surveillance System (e.g. DSS, Hot Spot monitoring, etc.) ……………………….,……………......3<br>Administrative (routine) data source (e.g. DHIS-2, HMIS, other administrative data) ………………..4<br>Other (please specify) ……………………………………………………………...……………….....5<br>I don't know ……………………………………………………………...………………..................6	
B2	In the last 12 months have you accessed or used data related to any of the following measures of nutritional status? Select all that apply.	**No - I have not accessed or used any data on nutritional status measures ………………………...0**<br><br>**Under-5:**<br>Wasting / WHZ …………………………………………………………...………………............1<br>Wasting / MUAC ………………………………………………………….………………..........2<br>Stunting / HAZ ………………………………………………………………...……………….........3<br>Overweight (WHZ or percentile) ……………………………………………………………...……..4<br>Underweight / WAZ ………………………………………………………...……………………....5<br>Low-birth weight (LBW) ……………………………………………………………..……………..6<br>Global /Moderate Acute Malnutrition (Classified by MUAC or WHZ) ………………………………7<br>Severe Acute Malnutrition (classified by MUAC, WHZ and/or oedema) ……………………………..8<br>Anemia (classified by hemoglobin) …………………………………………………………...…..9<br>Vitamin A deficiency ……………………………………………………………...………………..10<br>Other micronutrient deficiencies in under 5 (specify) …………………………………………………...11<br><br>**School-age children:**<br>Overweight …………………………………………………………...………………...............12<br>Anemia …………………………………………………………...………………......................13<br><br>**Adolescents (male or female):**<br>Underweight …………………………………………………………...………………..............14<br>Overweight …………………………………………………………...………………...............15<br>Anemia (classified by hemoglobin) …………………………………………………………...…16<br><br>**All adults 15-49 (male or female):**<br>Overweight or obesity / high BMI ………………………………………………………………..…..17<br>Diabetes …………………………………………………………...………………......................18<br>Hypertension …………………………………………………………...………………..............19<br><br>**Women of reproductive age (WRA):**<br>Short stature / stunting …………………………………………………………………..…………………20<br>Underweight / low BMI / low MUAC …………………………………………………………………...21	 <br><br><br><br><br><br>→B2a1<br><br><br><br>→B2b1<br><br><br><br><br><br><br><br>→B2b1<br>→B2b1<br>→B2b1<br><br><br>→B2h<br>→B2f1&B2j<br>→B2g1&B2k

		Anemia (classified by hemoglobin) ……………………………………………………………..…22<br><br>**Pregnant and lactating women:**<br>Underweight / low BMI /low MUAC …………………………………………………………....23<br>Night blindness ………………………………………………………...……………………..24<br>Anemia ……………………………………………………….....…………………................27<br>Iron deficiency ………………………………………………………...……………………...26	→B2e1
B2a1	From what types of data source did you access low-birth weight (LBW) data?<br>Please select all that apply	Household survey (e.g. DHS/MICS/SMART/other household survey) ……………………..…...1<br>Health facility survey (e.g. SPA, other) ……………………………………….....………………...2<br>Surveillance System (e.g. DSS, Hot Spot monitoring, etc.) ………………………,…………….....3<br>Administrative (routine) data source (e.g. DHIS-2, HMIS, other administrative data) ………………..4<br>Other (please specify) ……………………………………………………...………………...5	
B2a2	In your work context, which of these data sources are considered the "official" / most often quoted for low-birth weight (LBW) data?<br>Please select all that apply.	Household survey (e.g. DHS/MICS/SMART/other household survey) ……………………..…...1<br>Health facility survey (e.g. SPA, other) ……………………………………….....………………...2<br>Surveillance System (e.g. DSS, Hot Spot monitoring, etc.) ………………………,…………….....3<br>Administrative (routine) data source (e.g. DHIS-2, HMIS, other administrative data) ………………..4<br>Other (please specify) ……………………………………………………...………………...5	
B2a3	Are low-birth weight (LBW) data available at a frequency/interval that meets your needs?	Yes ………………………...………………………………………..……….............................1<br>No ……………………………………………………………………………………………2	→ B2a4
B2a4	How frequently would you prefer to have new low-birth weight (LBW) data available at a frequency/interval that meets your needs? for your purposes?	Every 6-10 years …………………………………………………….......……………….........1<br>Every 2-5 years ……………………………………………………….....……………….............2<br>Every year (annual) …………………………………………………..………………….......3<br>Quarterly …………………………………………………………………….......................4<br>Monthly ………………………………………………………....…………….....................5<br>Other: Please specify ……………………………………………………...………………....6	

B2b1	From what types of data source did you access low-birth weight (LBW) data?<br>Please select all that apply	Household survey (e.g. DHS/MICS/SMART/other household survey) ……………………………..…...1<br>Health facility survey (e.g. SPA, other) ……………………………………………...………………...2<br>Surveillance System (e.g. DSS, Hot Spot monitoring, etc.) ………………………..,……………......3<br>Administrative (routine) data source (e.g. DHIS-2, HMIS, other administrative data) ………………..4<br>Other (please specify) …………………………………………………………...………………...5	
B2l2	In your work context, which of these data sources are considered the “official” / most often quoted for vitamin A deficiency data?<br>Please select all that apply.	Household survey (e.g. DHS/MICS/SMART/other household survey) ……………………………..…...1<br>Health facility survey (e.g. SPA, other) ……………………………………………...………………...2<br>Surveillance System (e.g. DSS, Hot Spot monitoring, etc.) ………………………..,……………......3<br>Administrative (routine) data source (e.g. DHIS-2, HMIS, other administrative data) ………………..4<br>Other (please specify) …………………………………………………………...………………...5	
B2l3	Are vitamin A deficiency data available at a frequency/interval that meets your needs?	Yes ………………………...……………………………………..………...............................1<br>No ………………………………………………………………………………………………2	 → B2l4
B2l4	How frequently would you prefer to have new vitamin A deficiency data available at a frequency/interval that meets your needs? for your purposes?	Every 6-10 years ………………………………………………………...…………………..........1<br>Every 2-5 years ………………………………………………………...…………………............2<br>Every year (annual) ………………………………………………………...…………………......3<br>Quarterly ………………………………………………………...…………………........................4<br>Monthly ………………………………………………………...…………………..........................5<br>Other: Please specify ………………………………………………………...…………………....6	
B2b1	You identified you access data on adolescents, does this include younger children 10-14?	Yes ………………………...……………………………………..………...............................1<br>No ………………………………………………………………………………………………2	→ B2b2

B2b2	From what types of data source did you access adolescent data?<br>Please select all that apply	Household survey (e.g. DHS/MICS/SMART/other household survey) ………………………….....…...1<br>Health facility survey (e.g. SPA, other) ……………………………………………...………………...2<br>Surveillance System (e.g. DSS, Hot Spot monitoring, etc.) ………………………..,…………….....3<br>Administrative (routine) data source (e.g. DHIS-2, HMIS, other administrative data) ………………..4<br>Other (please specify) ………………………………………………………………...………………...5	
B2e1	From what types of data source did you access iron deficiency in pregnant and lactating women data?<br>Please select all that apply	Household survey (e.g. DHS/MICS/SMART/other household survey) ………………………….....…...1<br>Health facility survey (e.g. SPA, other) ……………………………………………...………………...2<br>Surveillance System (e.g. DSS, Hot Spot monitoring, etc.) ………………………..,…………….....3<br>Administrative (routine) data source (e.g. DHIS-2, HMIS, other administrative data) ………………..4<br>Other (please specify) ………………………………………………………………...………………...5	
B2e2	In your work context, which of these data sources are considered the "official" / most often quoted for iron deficiency in pregnant and lactating women data?<br>Please select all that apply.	Household survey (e.g. DHS/MICS/SMART/other household survey) ………………………….....…...1<br>Health facility survey (e.g. SPA, other) ……………………………………………...………………...2<br>Surveillance System (e.g. DSS, Hot Spot monitoring, etc.) ………………………..,…………….....3<br>Administrative (routine) data source (e.g. DHIS-2, HMIS, other administrative data) ………………..4<br>Other (please specify) ………………………………………………………………...………………...5	
B2e3	Are iron deficiency in pregnant and lactating women data available at a frequency/interval that meets your needs?	Yes ………………………...……………………………………………..………..............................1<br>No ……………………………………………………………………………………………………2	 → B2e4

B2e4	How frequently would you prefer to have new iron deficiency in pregnant and lactating women data available at a frequency/interval that meets your needs for your purposes?	Every 6-10 years ……………………………………………………………...……………….........1<br>Every 2-5 years ……………………………………………………………...……………….............2<br>Every year (annual) ……………………………………………………………...………………......3<br>Quarterly ……………………………………………………………...……………….......................4<br>Monthly ……………………………………………………………...………………........................5<br>Other: Please specify ……………………………………………………………...……………….....6	
B2f1	From what types of data source did you access diabetes data? Please select all that apply	Household survey (e.g. DHS/MICS/SMART/other household survey) ……………………………..…...1<br>Health facility survey (e.g. SPA, other) …………………………………………….....………………...2<br>Surveillance System (e.g. DSS, Hot Spot monitoring, etc.) ………………………..,……………......3<br>Administrative (routine) data source (e.g. DHIS-2, HMIS, other administrative data) ……………….4<br>Other (please specify) …………………………………………………………...………………...5	
B2f2	In your work context, which of these data sources are considered the "official" / most often quoted for diabetes data? Please select all that apply.	Household survey (e.g. DHS/MICS/SMART/other household survey) ……………………………..…...1<br>Health facility survey (e.g. SPA, other) …………………………………………….....………………...2<br>Surveillance System (e.g. DSS, Hot Spot monitoring, etc.) ………………………..,……………......3<br>Administrative (routine) data source (e.g. DHIS-2, HMIS, other administrative data) ……………….4<br>Other (please specify) …………………………………………………………...………………...5	
B2f3	Are diabetes data available at a frequency/interval that meets your needs?	Yes …………………………...……………………………………………..……….............................1<br>No ……………………………………………………………………………………………………2	→ B2f4
B2f4	How frequently would you prefer to have new diabetes data available at a frequency/interval that meets your needs for your purposes?	Every 6-10 years ……………………………………………………………...……………….........1<br>Every 2-5 years ……………………………………………………………...……………….............2<br>Every year (annual) ……………………………………………………………...………………......3<br>Quarterly ……………………………………………………………...……………….......................4<br>Monthly ……………………………………………………………...………………........................5<br>Other: Please specify ……………………………………………………………...……………….....6	

B2g1	From what types of data source did you access hypertension data? Please select all that apply	Household survey (e.g. DHS/MICS/SMART/other household survey) ……………………………..…...1<br>Health facility survey (e.g. SPA, other) …………………………………………...………………...2<br>Surveillance System (e.g. DSS, Hot Spot monitoring, etc.) ……………………….,……………......3<br>Administrative (routine) data source (e.g. DHIS-2, HMIS, other administrative data) ………………..4<br>Other (please specify) ……………………………………………………………...………………...5	
B2g2	In your work context, which of these data sources are considered the "official" / most often quoted for hypertension data? Please select all that apply.	Household survey (e.g. DHS/MICS/SMART/other household survey) ……………………………..…...1<br>Health facility survey (e.g. SPA, other) …………………………………………...………………...2<br>Surveillance System (e.g. DSS, Hot Spot monitoring, etc.) ……………………….,……………......3<br>Administrative (routine) data source (e.g. DHIS-2, HMIS, other administrative data) ………………..4<br>Other (please specify) ……………………………………………………………...………………...5	
B2g3	Are hypertension data available at a frequency/interval that meets your needs?	Yes ………………………...…………………………………………..………...............................1<br>No ……………………………………………………………………………………………………2	→ B2g4
B2g4	How frequently would you prefer to have new hypertension data available at a frequency/ interval that meets your needs for your purposes?	Every 6-10 years ………………………………………………………...……………….........1<br>Every 2-5 years ………………………………………………………..……………….............2<br>Every year (annual) …………………………………………………...……………….......3<br>Quarterly ……………………………………………………………...……………….......................4<br>Monthly ……………………………………………………………....……………….........................5<br>Other: Please specify …………………………………………………...…………………....6	
B2h	For which adult populations do you access overweight or obesity/ high BMI data?	Males ………………………………………………………...……………….............................1<br>Females ……………………………………………………...……………….........................2<br>Both males and females ……………………………………………………...………………3	

B2j	For which adult populations do you access diabetes data?	Males ……………………………………………………………...………………...........................1<br>Females …………………………………………………………...……………….........................2<br>Both males and females ………………………………………………………...………………3	
B2k	For which adult populations do you access hypertension data?	Males ……………………………………………………………...………………...........................1<br>Females …………………………………………………………...……………….........................2<br>Both males and females ………………………………………………………...………………3	
B3	In the last 12 months have you accessed or used data related to these infant and young child feeding practices? Select all that apply.	**No - I have not accessed or used any data on infant and young child feeding ……………………0**<br><br>***Breastfeeding:***<br>Early initiation of breastfeeding ……………………………………………………………..……..1<br>Exclusive breastfeeding (Up to 6m) ……………………………………………………...….2<br>Breastfeeding patterns (0-23m) (e.g. any, exclusive, predominant, etc.) ………………………………3<br>Duration of breastfeeding (e.g. in months, at 1 year, at 2 years, etc.) …………………………………4<br>Use of bottles ………………………………………………………...……………................5<br>Use of infant formula/ breastmilk substitute ……………………………………………………………6<br><br>***Complementary feeding (6-23 months):***<br>Dietary Diversity (e.g. Minimum Dietary Diversity- MDD; other food group scores) ………………..7<br>Consumption of specific food groups (e.g. iron-rock, animal source, vitamin a rich, etc.) ……………8<br>Frequency of feeding (e.g., Minimum Meal Frequency - MMF, other frequency score) ……………..9<br>Combine score of quality, frequency other feeding practices (e.g. Minimum Acceptable Diet – MAD, other feeding index) ……………………………………………………...………………....10<br>Age of Introduction of solid, semi-solid or soft foods ………………………………………………...11<br>Milk feeding frequency for non-breastfed children Complementary feeding food group intake ……..12	 <br><br>→B3a1<br>→B3a1<br>→B3a1<br>→B3a1<br>→B3a1<br>→B3a1<br><br><br>→B3a1<br>→B3a1<br>→B3a1<br><br>→B3a1<br>→B3a1<br>→B3a1
B3a1	From what types of data source did you access IYCF indicators? Please select all that apply	Household survey (e.g. DHS/MICS/SMART/other household survey) …………………………..…...1<br>Health facility survey (e.g. SPA, other) …………………………………………...………………...2<br>Surveillance System (e.g. DSS, Hot Spot monitoring, etc.) ……………………….,……………......3<br>Administrative (routine) data source (e.g. DHIS-2, HMIS, other administrative data) ………………..4<br>Other (please specify) ……………………………………………………………...………………...5	
B3a2	In your work context, which of these data sources are considered the "official" / most often quoted for IYCF indicators? Please select all that apply.	Household survey (e.g. DHS/MICS/SMART/other household survey) …………………………..…...1<br>Health facility survey (e.g. SPA, other) …………………………………………...………………...2<br>Surveillance System (e.g. DSS, Hot Spot monitoring, etc.) ……………………….,……………......3<br>Administrative (routine) data source (e.g. DHIS-2, HMIS, other administrative data) ………………..4<br>Other (please specify) ……………………………………………………………...………………...5	

B3a3	Are new IYCF data at a frequency/interval that meets your needs?	Yes …………………………...…………………………………………..………...............................1<br>No ……………………………………………………………………………………………………2	 → B3a4
B3a4	How frequently would you prefer to have new IYCF data available at a frequency/interval that meets your needs for your purposes?	Every 6-10 years ………………………………………………………………...……………….........1<br>Every 2-5 years ………………………………………………………………..……………….............2<br>Every year (annual) …………………………………………………………...…………………......3<br>Quarterly ……………………………………………………………………...…………………........................4<br>Monthly ……………………………………………………………………...………………….........................5<br>Other: Please specify …………………………………………………………...…………………....6	
B4	In the last 12 months have you accessed or used any data related to population-level hunger or food security status?	Yes …………………………...…………………………………………..………...............................1<br>No ……………………………………………………………………………………………………2	 →B5
B4b	Which of the following, if any, of the food security indicators have you accessed or used in the past 12 months? Select all that apply.	Prevalence of undernourishment (FAO) ………………………………………………...…………………..1<br>HFIAS (Household Food Insecurity and Access Scale) …………………………………………….....2<br>HFIES Household Food Insecurity Experience Scale (Gallup World Poll / FAO Voices of Hungry …3<br>HHS (Household Hunger Scale) …………………………………………...……………….............4<br>FCS (Food consumption Scores) …………………………………………...……………….............5<br>Proportion of expenditure on food …………………………………………...……………….........6<br>CSI (Coping Strategies Index) …………………………………………...………………….................7<br>Other (specify) …………………………………………...…………………...8	
B5	In the last 12 months have you accessed or used data related to diet quality in adults and/or at household level? Select all that apply.	**No - I have not accessed or used any data on diet quality in adults or households ………………0**<br><br>Women-specific dietary diversity (e.g. MDD-W, WDDS, other score) ………………………………..1<br>Household-level dietary diversity (e.g. HDDS, other index) ……………………………………..2<br>Any group: Intake of specific food groups (e.g. fruits and vegetable, animal source foods, etc.) ……..3<br>Any group: Sodium intake …………………………………………...…………………......................4<br>Any group: Consumption of unhealthy foods (e.g. sugar sweetened beverages, fatty foods, sugary foods) …………………………………………...…………………..5	

B6	In the last 12 months have you accessed or used data related to nutrition-sensitive interventions or drivers? Select all that apply.	**No - I have not accessed or used any data on nutrition sensitive interventions or determinants ..0**<br><br>**WASH**<br>Access to drinking water (e.g. safe, improved, accessible, etc.) ……………………………………….1<br>Access to toilet/latrine (e.g. safe, improved, etc.) …………………………………………………….2<br>Access to handwashing facilities ……………………………………………………………………….3<br>Hygiene practices (e.g. handwashing behavior, disposal of stools, etc.) ……………………………….4<br><br>**Health**<br>Antenatal care …………………………………………………………………………………………..5<br>Delivery (e.g. skilled birth attendants, facility delivery) ………………………………………………6<br>Immunizations in children ……………………………………………………………………………..7<br>Kangaroo mother care ………………………………………………………………………………….8<br>Malaria prevention (e.g. IPTP, ITNs, indoor spraying) ………………………………………………..9<br>Availability of health workers (e.g. density) ……………………………………………………………10<br><br>**Education**<br>Level of education (e.g. by gender) ……………………………………………………………………...11<br><br>**Family planning**<br>Use of Family Planning …………………………………………………………………………………12<br>Adolescent pregnancies or births ……………………………………………………………………...13<br><br>**Gender**<br>Gender Inequality (e.g. index) …………………………………………………………………………...14<br>Income, disaggregated by gender ……………………………………………………………………...15<br>Women's Empowerment in Agriculture Index (WEAI) ………………………………………………16<br>Women's time use and labour …………………………………………………………………………...17<br><br>**Agriculture**<br>Home/kitchen gardens …………………………………………………………………………………..18<br>Production of specific crops ……………………………………………………………………………19<br>Production of specific animals …………………………………………………………………………..20<br>Use of irrigation / water technology ……………………………………………………………………21<br>Use of other improved agriculture practices ……………………………………………………………22<br>Reach by agricultural extension agent …………………………………………………………………..23<br><br>**Social Protection**<br>Participation in cash transfer / safety net program ……………………………………………………24	

	**Section C: Data sources used**<br>*By data sources we mean source in any format that provides statistics relating to population nutritional status (anthropometry, micronutrient, etc.), behaviors (IYCF) and/or intervention coverage (vitamin A supplementation))*		
C1	In the last 12 months, which of the following NATIONAL data sources have you accessed / used from a report, dataset or other format? Select all that apply.	**National Household surveys:**<br>Demographic Health Survey (DHS) ……………………………………………………………….1<br>Multiple Indicator Cluster Survey (MICS) ……………………………………………………..2<br>National survey using SMART methodology ……………………………………………………..3<br>National Dietary Intake / Food Consumption Survey ……………………………………………..4<br>Other National Nutrition Survey (e.g. micronutrient survey) ……………………………………..5<br>World Bank Living Standard Measurement Studies(LSMS) ………………………………………..6<br>WFP Comprehensive Food Security and Vulnerability Assessments (CFSVA) ……………………………7<br>WFP Crop and Food Security Assessment Mission (CFSAM) ………………………………………...8<br>WFP Emergency Food Security Assessment (EFSA) …………………………………………………9<br>Other national household surveys with nutrition data (specify all name(s)) ……………………………..10<br><br>**Subnational Household Surveys:**<br>Sub-national survey using SMART methodology ………………………………………………………11<br>Other survey specific to program or policy-(please specify all others used) ……………………………..12<br><br>**Health facility survey:**<br>Service Provision Assessment (SPA) ……………………………………………………………….13<br>Other facility surveys (please specify all others used) …………………………………………………...14<br><br>**National monitoring /surveillance systems:**<br>Demographic surveillance sites (DSS) ………………………………………………………………..15<br>National food security "hot spot" monitoring system / FEWS-NET ……………………………………16<br>WFP Food Security Monitoring System (FSMS) (e.g. mVAM monitoring/Food Security Bulletins) …….17<br>Other national surveillance system (specify) …………………………………………………………18<br><br>**National administrative systems:**<br>DHIS-2 / similar online HMIS portal …………………………………………………………………19<br>Health Management Information System (HMIS) (not web-based portal) ……………………………….20<br>Agriculture sector MIS ………………………………………………………………………………...21<br>WASH sector MIS …………………………………………………………………………….........23<br>Education sector MIS ……………………………………………………………………………….....22<br>Other sector data systems (please specify all others used) …………………………………………….23<br><br>Other (please specify) …………………………………………………………………………….....24	

C2	In the last 12 months, which of the following GLOBAL consolidated data sources have you accessed? Please select all that apply.	**Global reports/profile:**<br>Countdown to 2030 (website/reports/country profiles) ……………………………………………………..1<br>Global Nutrition Report (website/reports/country profiles) ……………………………………………………2<br>Scaling up Nutrition MEAL (website/reports/country profiles) ……………………………………………..3<br>World Bank Nutrition Country Profiles (website/reports/country profiles) ………………………………..4<br>FAO The State of Food security and Nutrition in the World ……………………………….........................5<br>Hunger and Nutrition Commitment Index Global: Country profiles ……………………………..............6<br>UNICEF State of the World's Children Report Dashboard ………………………………........................7<br>WHO Global targets tracking tool ………………………………...8<br><br>**Global Databases:**<br>WHO Global Health Observatory ………………………………...9<br>UNICEF, WHO and the World Bank Joint Malnutrition Estimates / JME Dashboard ……………………10<br>Other UNICEF Nutrition datasets for specific topics (Vitamin A, iodine, low birthweight, IYCF) ……….11<br>WHO/UNICEF JMP (Joint Monitoring Programme for Water Supply, Sanitation and Hygiene) …………12<br>FAO/WHO GIFT (Global Individual Food consumption data Tool) ……………………………...........13<br>FAO Country Indicators ………………………………..14<br>WHO Vitamin & Mineral Nutrition Information Systems ………………………………..........................15<br>IHME Global Burden of Disease Comparison ………………………………...16<br>IHME Child Growth Failure ………………………………..17<br><br>Other (please specify) ………………………………..18	
	Section D: Indicators missing generally		
E1	Thinking about the countries / contexts where you work, are there any types of nutrition data and/or specific indicators that you want to access or use but are not available?<br><br>Please list/describe by category	Intervention Coverage [FREE RESPONSE]<br>Nutritional Status [FREE RESPONSE]<br>IYCF Practices [FREE RESPONSE]<br>Diet quality in adults or household [FREE RESPONSE]<br>Food Security or Hunger [FREE RESPONSE]<br>Nutrition-sensitive or other determinants [FREE RESPONSE]<br>Other [FREE RESPONSE]	
	Section F: Follow-up on data usability		
	Please select the challenges you currently experience in accessing and using data to support your work in nutrition. Please answer based on how frequently you experience these challenges. If you do not experience the challenge, please mark "Do not experience."		
F1a	Data is not analyzed or	Frequently experience ………………………………..1	

	visually presented so I find it difficult to interpret	Sometimes experience ....2<br>Rarely experience ....3<br>Do not experience 4	
F1b	Data is analyzed or visually presented but I still find it difficult to interpret and translate into actionable takeaways	Frequently experience ....1<br>Sometimes experience ....2<br>Rarely experience ....3<br>Do not experience 4	
F1c	There are multiple statistics and definitions listed for the same indicator so I am unsure which one to reference	Frequently experience ....1<br>Sometimes experience ....2<br>Rarely experience ....3<br>Do not experience 4	
F1d	Data is often out-of-date so I cannot use data to make decisions as frequently as I'd like	Frequently experience ....1<br>Sometimes experience ....2<br>Rarely experience ....3<br>Do not experience 4	
F1e	Data is not available at the geographical level I need (i.e., subnational)	Frequently experience ....1<br>Sometimes experience ....2<br>Rarely experience ....3<br>Do not experience 4	
F1f	Data is not available for the demographic group I need (i.e., sex, age, educational level, socioeconomic status)	Frequently experience ....1<br>Sometimes experience ....2<br>Rarely experience ....3<br>Do not experience 4	
F1g	Trend data does not exist / is not easily accessible so I am not clear on progress	Frequently experience ....1<br>Sometimes experience ....2<br>Rarely experience ....3<br>Do not experience 4	
F1h	Data quality cannot be trusted / is unreliable	Frequently experience ....1<br>Sometimes experience ....2<br>Rarely experience ....3<br>Do not experience 4	
F1i	The indicators I need do not have data	Frequently experience ....1<br>Sometimes experience ....2<br>Rarely experience ....3<br>Do not experience 4	
F1j	Presented data is not	Frequently experience 1	

	adequately summarized (e.g. no 95% CI's)	Sometimes experience ……………………………………2<br>Rarely experience ……………………………………3<br>Do not experience ……………………………………4	
F1k	Data is not available in raw format	Frequently experience ……………………………………1<br>Sometimes experience ……………………………………2<br>Rarely experience ……………………………………3<br>Do not experience ……………………………………4	
F1l	I am not sure which of the potential data sources is most appropriate for my needs	Frequently experience ……………………………………1<br>Sometimes experience ……………………………………2<br>Rarely experience ……………………………………3<br>Do not experience ……………………………………4	
	Section G: Potential for Follow-Up		
G1	To further the goal of improving the usability of nutrition data, our research team would greatly appreciate the opportunity to follow up with some survey respondents to better understand how they are using data. If you are willing to speak with us further about this topic, please leave your name and email where our team can reach you.<br><br>If you are not comfortable, that is okay.		
